# Cross-Activation of Regulatory T Cells by Self Antigens Limits Self-Reactive and Activated CD8^+^ T Cell Responses

**DOI:** 10.3390/ijms241813672

**Published:** 2023-09-05

**Authors:** Eunjung Cho, Seongeun Han, Hyeon Seok Eom, Sang-Jin Lee, Chungyong Han, Rohit Singh, Seon-Hee Kim, Bo-Mi Park, Byoung-Gie Kim, Young H. Kim, Byoung S. Kwon, Ki Taek Nam, Beom K. Choi

**Affiliations:** 1 Severance Biomedical Science Institute, Graduate School of Medical Science, Brain Korea 21 Project, Yonsei University College of Medicine, Seoul 03722, Republic of Korea; 2 Immuno-Oncology Branch, Division of Rare and Refractory Cancer, National Cancer Center, Goyang 10408, Republic of Korealeesj@ncc.re.kr (S.-J.L.);; 3 Hematological Malignancy Center of the Hospital, National Cancer Center, Goyang 10408, Republic of Korea; 4 Department of Cancer Biomedical Science, Graduate School of Cancer Science and Policy, National Cancer Center, Goyang 10408, Republic of Korea; 5 Department of Biomedical Laboratory Science, Catholic Kwandong University, Gangneung 25601, Republic of Korea; 6 Biomedicine Production Branch, Research Institute, National Cancer Center, Goyang 10408, Republic of Korea; 7 Department of Obstetrics and Gynecology, Samsung Medical Center, Sungkyunkwan University School of Medicine, Seoul 06351, Republic of Korea; 8 Eutilex, Co., Ltd., Geumcheon-gu, Seoul 08594, Republic of Korea; 9 Innobationbio, Co., Ltd., Mapo-gu, Seoul 03929, Republic of Korea

**Keywords:** peripheral tolerance, regulatory T cells, CD8^+^ T cell, self-antigen, p53

## Abstract

The interaction between regulatory T (Treg) cells and self-reactive T cells is a crucial mechanism for maintaining immune tolerance. In this study, we investigated the cross-activation of Treg cells by self-antigens and its impact on self-reactive CD8^+^ T cell responses, with a focus on the P53 signaling pathway. We discovered that major histocompatibility complex (MHC) I-restricted self-peptides not only activated CD8^+^ T cells but also induced the delayed proliferation of Treg cells. Following HLA-A*0201-restricted Melan-A-specific (pMelan) CD8^+^ T cells, we observed the direct expansion of Treg cells and concurrent suppression of pMelan^+^CD8^+^ T cell proliferation upon stimulation with Melan-A peptide. Transcriptome analysis revealed no significant alterations in specific signaling pathways in pMelan^+^CD8^+^ T cells that were co-cultured with activated Treg cells. However, there was a noticeable upregulation of genes involved in P53 accumulation, a critical regulator of cell survival and apoptosis. Consistent with such observation, the blockade of P53 induced a continuous proliferation of pMelan^+^CD8^+^ T cells. The concurrent stimulation of Treg cells through self-reactive TCRs by self-antigens provides insights into the immune system’s ability to control activated self-reactive CD8^+^ T cells as part of peripheral tolerance, highlighting the intricate interplay between Treg cells and CD8^+^ T cells and implicating therapeutic interventions in autoimmune diseases and cancer immunotherapy.

## 1. Introduction

The immune system relies on central tolerance mechanisms in the thymus to eliminate the majority of self-reactive T cells during development [1,2]. However, despite this rigorous process, a significant number of self-reactive T cells with low affinity for self-antigens manage to evade elimination and persist in the peripheral circulation [3]. Studies by Mark M. Davis and colleagues have shown that the frequency of CD8^+^ T cells recognizing endogenous peptides is comparable to that of naïve CD8^+^ T cells recognizing foreign antigens [4], and similar observations have been made in CD4^+^ T cells from healthy individuals [5]. These findings indicate that central tolerance mechanisms are not thoroughly effective in eliminating all self-reactive T cells. The presence of these self-reactive T cells highlights the ongoing challenge of achieving complete self-tolerance in clinical settings and simultaneously emphasizes the importance of peripheral tolerance mechanisms in regulating the activation and anergy of self-reactive T cells.

Peripheral tolerance refers to the mechanisms that maintain immune self-tolerance and prevent excessive activation of T cells outside the thymus [6]. Among the key players in peripheral tolerance, we can primarily rely on regulatory T (Treg) cells, which play a crucial role in suppressing immune responses and maintaining immune homeostasis [7]. Treg cells emerge not only from maturing T cells within the thymus gland but also from extrathymic sources, particularly within the gut. The development of both thymic and peripheral Treg cells relies on autoimmune regulator (AIRE)^+^ antigen-presenting cells (APCs), such as medullary thymic epithelial cells (mTECs) within the thymus and extrathymic AIRE-expressing cells (eTACs or Janus cells) and group 3 innate lymphoid cells (ILC3) located outside the thymus [8]. Notably, peripheral Treg cells in the gut play a pivotal role in balancing quick responses to dangerous disease-causing agents with the need for tolerance towards the microbiota. Recent research, encompassing three studies, reveals that the responsibility for the development of peripheral Treg cells [9,10,11] lies with RORγt-expressing APCs rather than conventional dendritic cells (DCs).

Treg cells exert their suppressive function through various mechanisms, including the production of immunosuppressive cytokines such as IL-10 and TGF-beta, as well as direct cell–cell contact-mediated suppression [7]. In the context of antigen-specific activity, Treg cells are known to inhibit the activation and proliferation of effector T cells by directly suppressing their activation and their suppressor function in the no antigen-specific manner, once they are activated [12]. Treg cells enable to modulate the activity of antigen-presenting cells and as a result, alter the local immune microenvironment, further contributing to immune suppression [13]. In addition, Treg cells suppress tumor-specific CD8 T cell cytotoxicity via TGF-beta signals [14]. However, it needs to be noted that Treg cells do not employ a universal suppression mechanism. One of the critical functions of Treg cells is to prevent the activation of naive T cells and to maintain immune tolerance by increasing the activation threshold of naive conventional T cells [15], requiring stronger stimulatory signals for their activation and proliferation [16]. This mechanism leads to the prevention of the unwarranted activation of self-reactive T cells and the development of autoimmune responses.

Various mechanisms have been reported to contribute to the suppressive function of Treg cells in maintaining immune tolerance [16]. However, the specific molecular mechanisms underlying peripheral tolerance following the activation of self-reactive CD8^+^ T cells remain incompletely understood [17,18,19,20]. Treg cells have been found to possess a diverse repertoire of self-reactive T cell receptors (TCRs) [21] and to be activated in an antigen-specific manner, suppressing activated conventional T cells with no antigen-specificity. Therefore, it seems that these self-reactive TCRs enable Treg cells to recognize self-antigens and exert suppressive effects on the activated self-reactive T cells.

In this study, when self-peptides derived from human telomerase reverse transcriptase (hTERT) and Wilms’ tumor 1 (WT-1), restricted by major histocompatibility complex (MHC) I, was used to expand self-peptide-specific CD8^+^ T cells from with peripheral blood mononuclear cells (PBMCs) of solid cancer patients, these peptides not only activated human CD8^+^ T cells but also induced cross-activation of human CD4^+^ T cells, including Treg cells. Further investigation using Melan-A-specific CD8^+^ T cells from healthy human volunteers revealed that MHC class I-restricted self-peptides induced a delayed proliferation of Treg cells via cross-activation of Treg cells and led to the suppression of activated self-reactive CD8^+^ T cells. This novel finding suggests a mechanism contributing to peripheral tolerance for activated self-reactive CD8^+^ T cells in autoimmune and cancer circumstances.

## 2. Results

### 2.1. MHC Class I-Restricted Peptides Not Only Activate CD8^+^ T Cells but Also Cross-Activate CD4^+^ T Cells, including Foxp3^+^ Treg Cells

**Figure 1:**
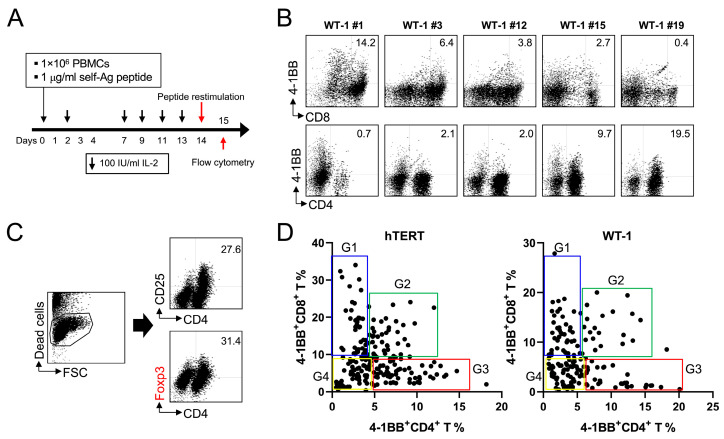
The proliferation of both CD8^+^ T cells and Treg cells in response to CD8^+^ T cell-restricted peptides derived from self-tumor antigens in vitro. (**A**) A schematic diagram illustrating the culture of peripheral blood mononuclear cells (PBMCs) with self-antigen peptides. (**B**) The representative result shows the 4-1BB expression of CD4^+^ T cells and CD8^+^ T cells following WT-1 peptide stimulation. The cultured PBMCs on day 15 were labeled with fluorescence-conjugated antibodies against CD4, CD8, and 4-1BB, and the expression of 4-1BB on CD4^+^ T cells and CD8^+^ T cells was measured using flow cytometry. (**C**) The cultured PBMCs on day 15 were stained with fluorescence-conjugated antibodies against CD4 and CD25, followed by fixation, permeabilization, and staining with fluorescence-conjugated anti-Foxp3 antibody to detect Treg cells. (**D**) Correlation between the percentage of 4-1BB-positive cells in CD4^+^ T cells and CD8^+^ T cells stimulated with hTERT and WT-1 peptides. Categorization was performed as follows: Group 1 (G1) encompassed peptides inducing 4-1BB solely on CD8^+^ T cells, Group 2 (G2) included peptides prompting 4-1BB expression on both CD8^+^ T and CD4^+^ T cells, Group 3 (G3) covered peptides exclusively inducing 4-1BB on CD4^+^ T cells, and Group 4 (G4) consisted of peptides failing to elicit 4-1BB activation in either CD8^+^ T or CD4^+^ T cells. These data represent a compilation of results from five independent analyses using blood samples from cancer patients.

For adoptive cell therapy using autologous CD8^+^ T cells targeting hTERT or WT-1, we selected 38 CD8^+^ T cell-restricted peptides (epitopes) against hTERT and 20 against WT-1 (Supplemental Tables S1 and S2). Because 4-1BB is predominantly induced on activated CD8^+^ T cells, it can function as a surrogate marker for peptide-specific CD8^+^ T cells (~). As a result, a procedure called “4-1BB-based isolation and expansion of peptide-specific CD8^+^ T cells” was established [22] and applied to produce CD8^+^ T cells specific to EBV, hTERT, and WT-1 for clinical use. As we previously reported [22], all patients did not equally respond to all selected peptides, and the response magnitude varied among patients. Therefore, before the production of hTERT- or WT-1-specific CD8^+^ T cells for clinical trials, each patient was tested to select peptides that would induce the expansion of CD8^+^ T cells (Figure 1A), which was assessed by detecting 4-1BB on CD8^+^ T cells (Figure 1B). Eventually, a mixture of four different peptides was used to produce hTERT- or WT-1-specific CD8^+^ T cells for clinical trials.

We conducted the screening test using PBMCs from 142 cancer patients to identify hTERT epitopes (Cytotherapy, accepted) and from approximately 200 cancer patients to determine WT-1 epitopes (manuscript in preparation) during the clinical trials and found one consistent pattern in the results—an inverse correlation between 4-1BB^+^CD8^+^ T cells and CD4^+^ T cells and/or 4-1BB^+^CD4^+^ T cells (Figure 1B). Since 4-1BB^+^CD4^+^ T cells increased even after the addition of MHC class I-restricted peptides, it was hypothesized that the peptides might induce the expansion of regulatory CD4^+^ T cells with self-reactive TCRs [23]. As expected, the PBMCs cultured, as shown in Figure 1A, including 4-1BB^+^CD4^+^ T cells, were intracellularly stained with anti-Foxp3 mAb, and Foxp3 was readily detected in the CD4^+^.

T cells (Figure 1C). During the phase I clinical trial involving hTERT- and WT-1-specific CD8^+^ T cells, approximately 43% of patients exhibited hTERT-reactive CD8^+^ T cells (61 patients out of 142 cancer patients), and 30–35% displayed WT-1-specific CD8^+^ T cells (data not shown). Consequently, we randomly selected five patients with both hTERT- and WT-1-specific CD8^+^ T cells, and the correlation between 4-1BB^+^CD8^+^ T cells and 4-1BB^+^CD4^+^ T cells was plotted five times. The consistency of the correlation results across these repetitions was evident, leading us to present representative data in Figure 1D.

The data showed that MHC class I-restricted peptides from hTERT and WT-1 have four different functional properties in inducing T cell responses—group 1 (G1) to group 4 (G4) (Figure 1D). We classified the hTERT or WT-1 peptides into four groups (G1–G4) based on their T cell responses, but the peptide types of the four groups were not consistent between different patients (data not shown). This inconsistency suggests that MHC class II subtypes were responsible for the response of CD4^+^ T cells to each self-peptide. Collectively, these data indicate that CD8^+^ T cell-restricted peptides originating from self-tumor antigens like hTERT and WT-1 activate not only self-reactive CD8^+^ T cells but also are likely to cross-activate CD4^+^ T cells, including Foxp3^+^ Treg cells with self-reactive TCRs.

### 2.2. Foxp3^+^ Treg Cells Are Expanded by Melan-A Self-Peptide, but Not by Non-Self CMV Peptide

**Figure 2:**
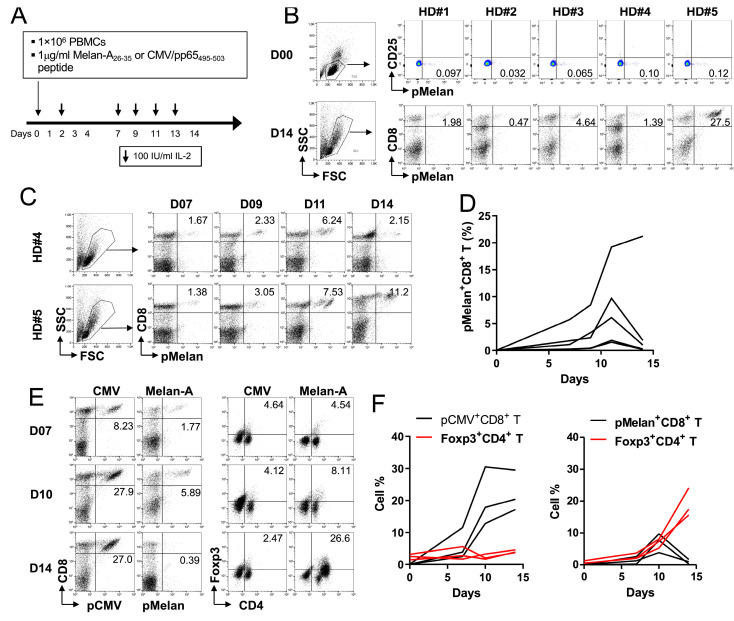
The proliferation of Foxp3^+^CD4^+^ T cells from peripheral blood mononuclear cells (PBMCs) in response to CTL peptides derived from melan-A. (**A**) A schematic diagram depicting the culture of PBMCs with CMV or Melan-A peptides. (**B**) Freshly isolated PBMCs from five healthy donors were stained with FVS780, anti-CD3, anti-CD8, anti-CD25, and A2/Melan-A_26-35_ multimer (pMelan). Live, and CD3^+^CD8^+^ cells were gated and plotted on a CD25 vs. pMelan. The PBMCs were cultured for 14 days as described in (**A**) and stained with anti-CD8 and A2/Melan-A_26-35_ multimer. (**C**) Kinetics of pMelan^+^CD8^+^ T cell frequencies during the 14-day culture. (**D**) Frequencies of pMelan^+^CD8^+^ T cells calculated from 5 healthy donors. Frequencies of pCMV^+^ or pMelan^+^ CD8 T cells and Foxp3^+^CD4^+^ T cells at the indicated days. (**F**) Frequencies of CMV/pp65_495-503_-specific (pCMV^+^) or pMelan^+^CD8^+^ T cells and Foxp3^+^CD4^+^ T cells calculated from the data in (**E**). Data are from three independent experiments with 5 healthy donors.

Since the peptide-originating self-antigens cross-activated and expanded Foxp3^+^ CD4^+^ Treg cells (Figure 1C), it was investigated whether this was unique to WT-1/hTERT epitopes or a general feature of Treg cells responding to self-antigens. Melan-A/MART-1_26-25_ is a well-studied human tumor-associated antigen recognized by CD8^+^ T cells with HLA-A*0201, and Melan-A_26-25_-specific CD8^+^ T cells are found abundantly in healthy individuals and can be readily expanded by Melan-A_26-25_ peptide [24,25]. Additionally, the CMV/pp65_495–503_ peptide is also restricted to HLA-A*0201. To investigate this further, we screened five healthy individuals (HD#1–#5) with the HLA-A*02 allele and the presence of Melan-A_26-35_ and CMV/pp65_495-503_-specific CD8^+^ T cells. Flow cytometric analysis showed that CD8^+^ T cells from these individuals had 0.032–0.12% of Melan-A_26-35_-specific CD8^+^ T cells that were inactivated (CD25-negative phenotype) (Figure 2B, upper panel). When PBMCs from these individuals were cultured in the presence of Melan-A peptide for 14 days and were stained with MHC class I/Melan-A_26-35_ multimer (HLA-A*0201-restricted; pMelan) along with anti-CD8-APC (Figure 2A), the percentages of pMelan^+^CD8^+^ T cells at day 14 varied (Figure 2B, lower panel).

The percentages of pMelan^+^CD8^+^ T cells at day 14 did not correlate with their initial frequencies (Figure 2B), prompting us to examine the expansion kinetics of pMelan^+^CD8^+^ T cells during the culture process. Melan-A_26-35_ peptide stimulation led to a steady increase in pMelan^+^CD8^+^ T cells until day 11, followed by a sharp decline, except for HD#5 (Figure 2C,D). The rapid decrease in pMelan^+^CD8^+^ T cells after day 11 was consistently observed in HD#1–4, except for HD#5 (Figure 2D). To determine whether this phenomenon was specific to self-peptide, we stimulated PBMCs from HD#1–3 with Melan-A_26-35_ or CMV/pp65_495-503_ peptide. CMV peptide induced a robust and continuous increase in pCMV/pp65^+^CD8^+^ T cells until day 14, while pMelan^+^CD8^+^ T cells peaked at day 10 and then started to decline. Foxp3^+^CD4^+^ Treg cells were also not increased by non-self CMV peptide but were elevated by Melan-A self-peptide, particularly during days 10–14 (Figure 2E).

This pattern, an inverse correlation between the decrease in pMelan^+^CD8^+^ T cells and the increase in Foxp3^+^CD4^+^ Treg cells, was consistently observed in all three donors (Figure 2F). These data suggest that self-peptides such as hTERT, WT-1, and Melan-A can stimulate both self-reactive CD8^+^ T cells and Foxp3^+^CD4^+^ Treg cells with self-reactive TCRs, whereas foreign antigens preferentially seem to activate conventional CD8^+^ T cells. Therefore, we conclude that the expansion of Foxp3^+^CD4^+^ Treg cells by self-antigens could be a general phenomenon.

### 2.3. Melan-A Self-Peptide Directly Expands Foxp3^+^ Treg Cells

**Figure 3:**
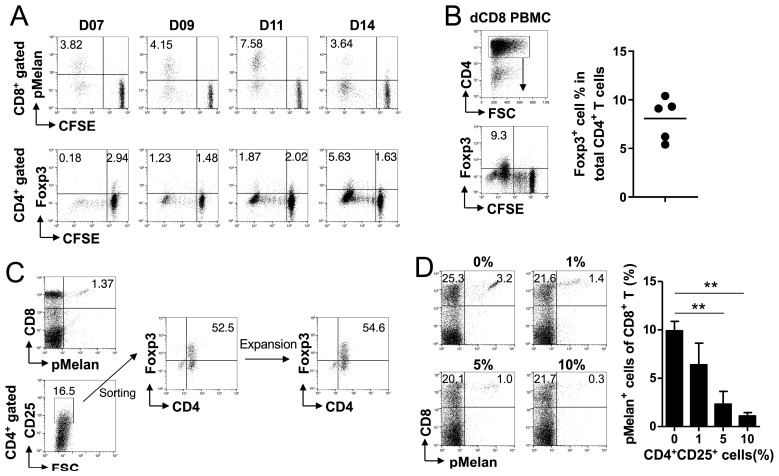
Suppression of Melan-A-specific CD8^+^ T cell proliferation by expanded Foxp3^+^CD4^+^ T cells. (**A**) CFSE-labeled PBMCs from pMelan^+^ healthy donors were cultured with Melan-A peptide and IL-2 for 14 days. The cultured PBMCs were analyzed by flow cytometry using anti-CD8 and MHC I/Melan-A multimer staining for gated CD8^+^ T cells and anti-CD4 and anti-Foxp3 intra-cellular staining for gated CD4^+^ T cells. (**B**) Freshly isolated PBMCs were depleted of CD8^+^ T cells and labeled with CFSE. The cells were cultured with Melan-A peptide and IL-2 for 14 days. The cultured cells were analyzed by flow cytometry using anti-CD4 and anti-Foxp3 intra-cellular staining. (**C**) PBMCs cultured with Melan-A peptide for 14 days were stained with anti-CD4 and anti-CD25 antibodies. CD4^+^CD25^High^ cells were sorted and expanded by the rapid expansion method. CD4^+^CD25^High^ cells before and after the expansion were analyzed by flow cytometry using anti-CD4 and anti-Foxp3 staining. (**D**) Freshly isolated PBMCs from pMelan^+^ healthy donors and expanded CD4^+^CD25^High^ cells, including approximately 50% Foxp3^+^CD4^+^ T cells, were mixed at different ratios and cultured with Melan-A peptide and IL-2. On day 10, cultured PBMCs were stained with anti-CD8 and MHC I/Melan-A multimer, and the percentages of pMelan^+^CD8^+^ T cells were determined by flow cytometry. Data are from two independent experiments with *n* = 3 in (**A**,**D**) and *n* = 5 in (**B**). Student’s *t*-test was performed in (**D**) and shown as the means ± SDs (** *p* < 0.01).

Although Melan-A_26-35_ peptide increased Foxp3^+^CD4^+^ Treg cells (Figure 2E,F), it was not clear whether Melan-A_26-35_ peptide directly triggered the activation of Foxp3^+^CD4^+^ Treg cells and whether the expanded Foxp3^+^CD4^+^ cells were Treg cells. When CFSE-labeled PBMCs from selected HDs were cultured in the presence of Melan-A peptide, pMelan^+^CD8^+^ T cells had already divided >9–10 times at day 7 in showing CFSE-negative population, steadily increasing until day 11, and then decreasing (Figure 3A; upper panel). Meanwhile, most of the Foxp3^+^CD4^+^ Treg cells had not divided until day 7 but vigorously divided between days 11 and 14 (Figure 3A; lower panel). In a separate experiment, CD8^+^ T cells were depleted before CFSE labeling of PBMCs and the CD8-depleted PBMCs were cultured in the presence of Melan-A peptide, and again most of the Foxp3^+^CD4^+^ cells were CFSE-negative at day 14, suggesting that Melan-A peptide directly activated Foxp3^+^CD4^+^ Treg cells and induced their division, particularly during days 11–14 (Figure 3B). In addition, when the isolated CD8^+^ T cells mixed with 10% CD14^+^ monocytes were cultured in the presence of Melan-A peptide for 14 days, the percentages of pMelan^+^CD8^+^ T cells among the isolated CD8^+^ T cells were considerably higher than those observed in the PBMC culture on day 14 (Supplementary Figure S1). Foxp3 staining indicates that Foxp3^+^CD4^+^ Treg cells were markedly decreased in CD8^+^ T and monocyte mixture compared with that PBMC culture (Supplemental Figure S1), which suggests that the expanded Treg cells were involved in the suppression of pMelan^+^CD8^+^ T cells.

In order to confirm if the expanded Foxp3^+^CD4^+^ Treg cells suppressed the proliferation of pMelan^+^CD8^+^ T cells, CD4^+^CD25^High^ T cells, including Foxp3^+^CD4^+^ Treg cells, were isolated on day 14 (Figure 3C) and were expanded by the rapid expansion method [26]. Intracellular staining of Foxp3 showed that the isolated CD4^+^CD25^High^ T cells contained ~50% of Foxp3^+^CD4^+^ Treg cells, which remained unchanged even after their expansion (Figure 3C). Freshly isolated PBMCs were then prepared from the same HD and were stimulated with Melan-A peptide for 2 days, and further were cultured with 0%, 1%, 5%, and 10% of the expanded Treg cells until day 10. Flow cytometry showed that pMelan^+^CD8^+^ T cells were readily detected in the absence of expanded Treg cells but disappeared in a dose-dependent manner in their presence (Figure 3D). These results suggest that Melan-A peptide directly activated Foxp3^+^CD4^+^ Treg cells and their proliferation was slower than that of pMelan^+^CD8^+^ T cells. Moreover, the expanded Foxp3^+^CD4^+^ Treg cells had the ability to suppress the activated pMelan^+^CD8^+^ T cells.

### 2.4. No Specific Signaling Pathway Is Induced in pMelan^+^CD8^+^ T Cells by Contact with Foxp3^+^ Treg Cells

**Figure 4:**
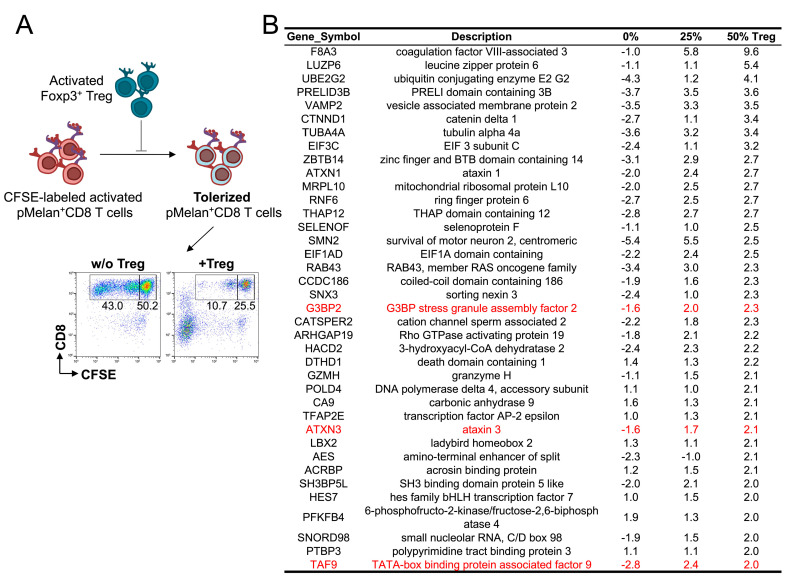
Upregulated mRNA expression in Melan-A-specific CD8^+^ T cells after co-culture with activated Foxp3^+^CD4^+^ T cells. PBMCs were stimulated with Melan-A peptide for 10 days, and pMelan^+^CD8^+^ T cells were sorted and expanded using the rapid expansion method. The expanded cells were rested for 5 days and then co-cultured with varying ratios of activated Foxp3^+^ Treg cells prepared, as shown in Figure 3C. (**A**) CFSE-labeled pMelan^+^CD8^+^ T cells were co-cultured with a 50% ratio of the expanded Treg cells for 5 days; the cells were stained with anti-CD8 antibody and analyzed by flow cytometry to assess CFSE dilution of CD8^+^ T cells. (**B**) Transcriptome analysis of pMelan^+^CD8^+^ T cells. Two days after the co-culture, pMelan^+^CD8^+^ T cells were isolated using CD8 microbeads. Total RNA was extracted from the isolated cells and subjected to RNA sequencing. The table lists the mRNAs that exhibited increased expression after exposure to activated Foxp3^+^ Treg cells.

In the extension of the observation that Melan-A peptide induced delayed-proliferation of Foxp3^+^CD4^+^ Treg cells (Figure 3A,B) and they suppressed activated pMelan^+^CD8^+^ T cells (Figure 3D), we investigated the mechanism by which activated Foxp3^+^CD4^+^ Treg cells suppress activated pMelan^+^CD8^+^ T cells. While Treg cells have been shown to suppress immune reactions via various mechanisms such as inhibitory ligands, suppressive cytokines, and direct killing of target cells [27], there is still no consensus on a universal mechanism. To address this, we analyzed transcriptome changes in activated pMelan^+^CD8^+^ T cells following their contact with Foxp3^+^CD4^+^ Treg cells. pMelan^+^CD8^+^ T cells and Foxp3^+^CD4^+^ Treg cells were isolated and expanded from a single donor, and the rested pMelan^+^CD8^+^ T cells were CFSE labeled and were stimulated with anti-CD3 mAb in the presence of 0%, 25%, and 50% unlabeled Foxp3^+^CD4^+^ Treg cells. CFSE dilution assay for 5 days showed that Foxp3^+^CD4^+^ Treg cells suppressed the proliferation of pMelan^+^CD8^+^ T cells (Figure 4A).

For transcriptome analysis, CD8^+^ T cells were isolated from the mixture of pMelan^+^CD8^+^ T cells and Foxp3^+^CD4^+^ Treg cells after 2 days of culture, and total RNAs were harvested for RNA sequencing, aiming to identify specific signaling pathways that were altered in CD8^+^ T cells upon contact with Treg cells. Far from the expectation, there were no significant changes in the signaling pathways (Supplementary Table S3). Therefore, we shifted our focus to the up-regulated genes in CD8^+^ T cells that were induced by Treg cells, as we hypothesized that these genes could potentially be involved in the suppression of pMelan^+^CD8^+^ T cells. Approximately 60 genes were up-regulated in pMelan^+^CD8^+^ T cells, with a fold increase in more than 2-fold in the presence of 50% Treg cells, as shown in Figure 4B. Although we did not observe a clear association between the up-regulated genes and apoptosis, three genes caught our attention: G3BP2 (G3BP stress granule assembly factor 2), ATXN3 (ataxin 3), and TAF9 (TATA-box binding protein associated factor 9). These genes are known to be involved in P53 degradation, and P53 plays a crucial role in inducing apoptosis in both transformed and non-transformed cells, primarily via the pro-apoptotic factors PUMA and/or NOXA [28]. G3BP2 reduces MDM2-mediated P53 ubiquitylation and degradation [29], ATXN3 stabilizes P53 via deubiquitination [30], and TAF9 prevents MDM2-mediated degradation of TP53 [31]. Furthermore, the accumulation of P53 results in apoptosis within activated CD8^+^ T cells [32], and inhibiting or lacking P53 significantly enhances glucose metabolism and the differentiation of CD8^+^ T cells with effector functions [33,34,35]. Hence, we raise the question of whether modulating P53 could potentially protect pMelan^+^CD8^+^ T cells from the suppressive activities exerted by Treg cells.

### 2.5. Pifithrin-α Hydrobromide Induces Continuous Growth of pMelan^+^CD8^+^ T Cells

**Figure 5:**
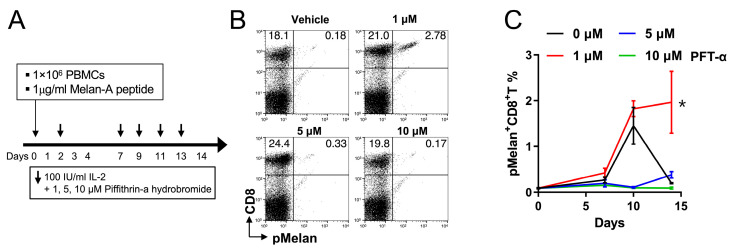
Pifithrin-α hydrobromide restores Treg-mediated suppression of activated pMelan^+^CD8^+^ T cells. (**A**) PBMCs were cultured with Melan-A peptide and IL-2, as described previously. The transcription of p53 was blocked by adding 0, 1, 5, or 10 μM of pifithrin-α hydrobromide (PFT-α) to the culture, as indicated. (**B**,**C**) The cultured PBMCs were stained with anti-CD8 antibody and MHC I/Melan-A multimer on days 0, 7, 10, and 14. (**B**) The percentages of pMelan^+^CD8^+^ T cells at day 14. (**C**) Kinetics of pMelan^+^CD8^+^ T cell frequencies during the culture. Representative data from two independent experiments is shown (*n* = 3). Student’s *t*-test was performed in C and shown as the means ± SDs (* *p* < 0.05).

To test the role of P53 on Treg cell-mediated suppression of activated CD8^+^ T cells, we investigated the impact of P53 blockade on Treg cell-mediated suppression by cultivating PBMCs from healthy donors with pMelan^+^CD8^+^ T cells in the presence of varying concentrations (0, 1, 5, and 10 μM) of pifithrin-α hydrobromide (PFT-α), a blocker of P53 transcriptional activity [27]. Flow cytometry analysis revealed that in the absence of PFT-α, the percentages of pMelan^+^CD8^+^ T cells sharply decreased between days 10 and 14. However, the treatment of pMelan^+^CD8^+^ T cells with 1 μM PFT-α rescued the decline in pMelan^+^CD8^+^ T cell percentages (Figure 5B,C). Higher concentrations of PFT-α (5 or 10 μM) showed a similar pattern to vehicle-treated PBMCs, likely due to the toxicity of PFT-α.

In a separate experiment, CFSE-labeled pMelan^+^CD8^+^ T cells were incubated with anti-CD3 monoclonal antibody and 1 μM PFT-α, both in the presence or absence of 50% Treg cells, for a duration of 5 days. Following the incubation, the gated pMelan^+^CD8^+^ T Cells were subjected to analysis for the proliferation index. The result revealed that Treg cells reduced the proliferation of pMelan^+^CD8^+^ T cells, which was reversed by the presence of PFT-α. Notably, the impact of PFT-α treatment on the proliferation of pMelan^+^CD8^+^ T cells alone was minimal (Supplemental Figure S2). These findings support that the blockade of P53-mediated transcriptional activity rescues pMelan^+^CD8^+^ T cells from suppression by activated Foxp3^+^CD4^+^ Treg cells.

## 3. Discussion

In this study, we serendipitously discovered that MHC I-restricted self-peptides derived from hTERT and WT-1 activated CD8^+^ T cells and cross-activated CD4^+^ T cells, including Treg cells (Figure 1). To confirm this observation, we stimulated pMelan^+^CD8^+^ T cells with HLA-A*0201-restricted Melan-A_26-35_ peptide, leading to Treg cell expansion and suppression of pMelan^+^CD8^+^ T cell proliferation (Figure 2 and Figure 3). Transcriptome analysis of pMelan^+^CD8^+^ T cells co-cultured with activated Treg cells did not reveal significant changes in specific signaling pathways (Figure 4). However, genes involved in P53 accumulation, a regulator of cell survival and apoptosis, were upregulated (Figure 4), and blocking P53 reversed Treg cell-mediated suppression of pMelan^+^CD8^+^ T cells (Figure 5). These findings demonstrate that MHC I-restricted self-peptides activate CD8^+^ T cells and induce delayed proliferation of Treg cells, and the concurrent stimulation of Treg cells via self-reactive TCRs by self-antigens represent natural mechanisms within the immune system, providing peripheral tolerance for activated self-reactive CD8^+^ T cells.

Treg cells play a critical role in maintaining peripheral tolerance by suppressing the activation of naive T cells via the modulation of T cell activation thresholds [16]. Numerous studies have reported the mechanisms by which Treg cells exert their suppressive effects on naive T cells [7]. However, there is a notable gap in our understanding of how Treg cells control the activation of already activated self-reactive CD8^+^ T cells. While the mechanisms underlying the suppression of naive T cells by Treg cells have been extensively investigated, the specific mechanisms employed by activated Treg cells to regulate activated self-reactive CD8^+^ T cells remain largely unexplored.

The activation and specificity of T cells in the immune system are regulated by MHC molecules [36]. CD8^+^ T cells recognize antigenic peptides that are 8 to 11 amino acids long and presented by MHC class I molecules, with specific anchor residues at positions 2 and 9 [37]. On the other hand, CD4^+^ T cells, including Treg cells, interact with antigenic peptides that are 12 to 25 amino acids long and displayed by MHC class II molecules, with anchor residues at positions 1, 4, 6, and 9 [38]. These distinct peptide lengths and anchor residue preferences play a critical role in the binding and presentation of peptides by MHC class I and class II molecules, respectively. The complementarity-determining region 1 and 2 (CDR1 and -2) loops of the TCR primarily interact with the conserved helical region of the MHC surface, while the more variable somatically rearranged CDR3 loops mainly contact the antigenic peptide [39,40]. However, a study by Cole et al. [41] provides an explanation of how MHC class I-restricted peptides with 8–11 amino acids can bind to MHC class II molecules that bind peptides of 12–25 amino acids. They demonstrate that CD8^+^ T cells specific for Melan-A_26-35_ predominantly express the TRAV12-2 gene for their TCRα chain. The CDR1 and CDR2 loops of their TCR are encoded by the TRAV12-2 and TRBV30 genes, respectively, and the specificity of their TCR for HLA-A2/Melan-A_26-35_ is primarily achieved via interactions with the CDR1α loop. This loop significantly contributes to peptide binding and functions similarly to classical CDR3 loops during MHC/peptide complex binding. The TRAV12-2 gene is also commonly found in CD4^+^ T cells [42,43], and the specificity of the TCR for HLA-A2/Melan-A_26-35_ is primarily achieved via interactions with the CDR1α loop. If the mild but specific binding of self-peptide to TCR is primarily determined via interaction with CDR1/2 rather than CDR3, as seen in the TCR for HLA-A2/Melan-A_26-35_, the length of the epitope may not be a limiting factor in self-epitope sharing between MHC class I and II. Therefore, further investigation is needed to determine whether self-epitope sharing between MHC class I and II molecules is a common feature among self-antigen epitopes.

In the context of self-reactive T cells, central tolerance mechanisms aim to remove or inactivate T cells with high-affinity TCRs toward self-antigens to prevent autoimmunity. As a result, self-reactive T cells generally exhibit low TCR affinities [36], and thus, self-peptides with low TCR affinity have a chance to cross-activate CD8^+^ T cells as well as CD4^+^ T and Treg cells, regardless of MHC class I/II restriction. Indeed, although we selected 38 hTERT and 20 WT-1 peptides restricted to MHC class I (Supplemental Tables S1 and S2), T-cell responses against the selected peptides varied among cancer patients (Figure 1B,D). Nevertheless, it was clear that the selected CD8^+^ T cell-restricted peptides not only activated CD8^+^ T cells in some patients but also induced the proliferation of CD4^+^ T cells, including Treg cells (Figure 1B–D), which suggests that every self-peptide with low TCR affinity has the potential to cross-activate Treg cells.

The current study primarily addressed the observed delayed proliferation of Treg cells. However, MHC I-restricted self-peptides derived from hTERT and WT-1 not only triggered the activation of Treg cells but also induced the proliferation of CD4^+^ T cells within the PBMCs of certain patients (Figure 1B). Considering that both conventional CD4^+^ T cells and Treg cells originate from the same thymic CD4^+^ T cell pool [12], it appears that conventional CD4^+^ T cells, especially those with self-reactivity, can be activated using these self-peptides. However, since activated Treg cells exert suppressive effects on both activated CD8^+^ T and CD4^+^ T cells, the mechanism of Treg cell-mediated suppression plays a crucial role in regulating self-reactive inflammation, even if CD4^+^ T cells are concurrently activated using self-peptides alongside CD8^+^ T cells.

Intrinsic differences in the proliferation rates of CD4^+^ and CD8^+^ T cells have been well-documented, with CD8^+^ T cells generally displaying a faster proliferation rate compared to CD4^+^ T cells [44]. As Treg cells arise from CD4^+^ T cell precursors [45,46], it is reasonable to expect that their proliferation rate would also be slower than that of CD8^+^ T cells. Consistent with this notion, our study demonstrated that stimulation of pMelan^+^ CD8^+^ T cells with Melan-A peptide resulted in their accelerated proliferation while concurrently leading to a delayed proliferation of Treg cells (Figure 2F and Figure 3A). Furthermore, the optimal suppressive activity of Treg cells requires activation via TCR stimulation [47]. Therefore, when naïve Treg cells are stimulated with self-antigen, they undergo delayed proliferation, acquire enhanced suppressive function, and start to suppress activated CD8^+^ T cells as the percentage of pMelan^+^CD8^+^ T cells decreases during days 10–11 (Figure 2 and Figure 3A). Although the activation and proliferation of Treg cells are antigen-specific, their suppressive effector function is not antigen-specific [12]. Consequently, when self-antigens released from damaged or abnormal cells simultaneously activate both self-reactive T cells, the concurrent activation of Treg cells by different or the same epitopes of self-antigens enables effective control of the activated self-reactive T cells by Treg cells equipped with potent suppressive functions.

In our study, transcriptome analysis of pMelan^+^CD8^+^ T cells in contact with activated Treg cells revealed increased expression of three genes—G3BP2, ATXN3, and TAF9, which are associated with P53 upregulation (Figure 4). P53 is involved in apoptosis and differentiation of activated CD8^+^ T cells [32,33,34,35], suggesting that modulation of P53 could potentially protect pMelan^+^CD8^+^ T cells from Treg cell-mediated suppression. Indeed, blocking P53-mediated transcriptional activity rescues pMelan^+^CD8^+^ T cells from suppression by activated Foxp3^+^CD4^+^ Treg cells (Figure 5 and Supplemental Figure S2). It is worth noting that a previous study has demonstrated that P53-deficient mouse CD8^+^ T cells exhibited enhanced glycolytic activity, increased proliferation, IFN-γ secretion, and cytotoxicity [33,34,35]. However, as PFT-α did not exclusively target the P53 of CD8^+^ T cells, additional investigation will be necessary to elucidate the mechanisms by which P53 modulation protects activated CD8^+^ T cells from Treg cell-mediated suppression.

The ultimate goal of cancer immunotherapy is to establish a self-sustaining immune response against cancer, encompassing CD8^+^ T cell responses against both self-tumor antigens and neo-antigens [48]. However, our observations highlight that the delayed proliferation of Treg cells prompted by self-tumor antigens can disrupt the continuity of the immune response against cancer. In this context, we provide evidence that MHC class I-restricted self-peptides induce the delayed proliferation of Treg cells, culminating in the suppression of activated self-reactive CD8^+^ T cells. However, it remains uncertain whether our findings can be universally applied as components of the peripheral tolerance mechanism for activated self-reactive CD8^+^ T cells. Regardless, the imperative arises to formulate strategies that efficaciously curtail the delayed proliferation of Treg cells or counterbalance the potent suppressive activity induced by self-antigen stimulation in activated Treg cells. The development of such approaches is indispensable for sustaining potent and enduring cancer immunotherapy against various types of cancers.

## 4. Materials and Methods

### 4.1. Reagents and Antibodies

All antibodies for flow cytometry, including anti-4-1BB-PE, anti-CD4-PE-Cy5, anti-CD8-APC, anti-CD8-PE-Cy5, anti-CD3-BV785, anti-CD25-PerCP-Cy5, anti-CD25-PE-Cy5, anti-CD4-APC, anti-CD25-FITC, fixable viability stain 780, and 7-AAD were obtained from BD Biosciences (San Jose, CA, USA). FoxP3/Transcription Factor Staining Buffer Set and functional grade anti-CD3 monoclonal antibody were from eBioscience (San Diego, CA, USA). CellTrace carboxyfluorescein succinimidyl ester (CFSE) was purchased from Thermofisher Scientific (Waltham, MA, USA). Recombinant human interleukin-2 was purchased from Novartis (Basel, Switzerland). HLA-A*0201-restricted cytomegalovirus (CMV)/pp65_495-503_ NLVPMVATV MHC class I multimer (pCMV/pp65) and HLA-A*0201-restricted Melan-A_26-35_ ELAGIGILTV MHC class I multimer (pMelan) were from Proimmune (Oxford, UK). All peptides, including hTERT and WT-1 peptides, CMV/pp65_495–503_, and Melan-A_26-35_, were chemically synthesized by Peptron (Daejeon, Republic of Korea). Pifithrin-α hydrobromide was from Santa Cruz Biotechnology (Santa Cruz, CA, USA).

### 4.2. Expansion of Peptide-Specific CD8^+^ T Cells from PBMCs

Blood samples were provided by healthy volunteers and cancer patients, including lung, ovarian, and cervical cancers, at least 4 weeks after the last treatment if the patient received chemotherapy or radiotherapy after obtaining written informed consent. The study was reviewed and approved by the Institutional Review Board of the National Cancer Center (NCCCTS13714, NCCCTS12629, and NCC2018-0093). Initially, five healthy donors (HDs) possessing HLA-A*0201-restricted CD8^+^ T cells specific to CMV/pp65_495–503_ and Melan-A_26-35_ (HD#1–#5) participated in this study. However, HD#4 and HD#5 were solely included in the assessment of the expansion kinetics of pCMV/pp65^+^ and pMelan^+^CD8^+^ T cells in Figure 2B,C. This decision was due to HD#5 not showing Treg cell-mediated suppression and HD#4’s preference not to participate in this study. Peptide-specific CD8^+^ T cells were expanded from PBMCs as described before [22]. In brief, PBMCs were isolated by centrifugation on a Ficoll density gradient and suspended in RPMI1640 medium (WelGENE, Seoul, Republic of Korea) supplemented with 4 mM L-glutamine, 12.5 mM HEPES, 50 mM 2-mercaptoethanol, and 3% auto plasma (CTL medium; CM) at 1 × 10^6^ cells/mL density, and 1 mL samples were placed in 15-mL round-bottomed tubes (BD Biosciences, San Jose, CA, USA). Cultures were maintained in a CO_2_ incubator with 2 μg/mL of antigenic peptides from hTERT, WT-1, CMV/pp65, or Melan-A for 2 days, after which 1 mL of fresh medium containing 100 IU/mL of rhIL-2 was added to each tube. Half of the medium in each tube was replaced with fresh medium supplemented with 100 IU/mL IL-2 on days 7, 9, 11, and 13.

### 4.3. Induction and Detection of 4-1BB on Peptide-Specific CD8^+^ T Cells

To induce 4-1BB on peptide-specific CD8^+^ T cells, the cultured PBMCs at day 14 were harvested, washed with RPMI1640 medium, and resuspended in CM at 2 × 10^6^ cells/mL density. The cells were cultured in a CO_2_ incubator with 5 μg/mL of the same antigenic peptide and 100 IU/mL of rhIL-2. After 24 h, the cells were harvested, washed with RIMP1640, and stained with anti-4-1BB-PE, anti-CD4-PE-Cy5, and anti-CD8-APC. All samples were subsequently analyzed by FACSCalbur (BD Bioscience).

### 4.4. Flow Cytometry

To assess the percentages of Melan-A-specific CD8^+^ T cells, freshly isolated PBMCs were first stained with Fixable Viability Stain 780, washed with RPMI1640 medium, and further stained with pMelan-PE, anti-CD3-BV785, anti-CD8-APC, and anti-CD25-PerCP-Cy5. Live CD3^+^CD8^+^ cells were gated and plotted pMelan vs. CD25. To detect the expanded CMV/pp65- and Melan-A-specific CD8^+^ T cells, the cultured PBMCs as described above were harvested at day 10 or 14, washed with RPMI1640 medium, and stained with pCMV/pp65-PE or pMelan-PE along with anti-CD8-PE-Cy5. To detect Foxp3^+^ Treg cells, the cultured PBMCs for 14 days, as described above, were harvested and washed with RPMI1640 medium. The cells were first stained with Fixable Viability Stain 780 and followed the surface staining of anti-CD25-FITC and anti-CD4-PE-Cy5. The stained cells were fixed and permeabilized with Foxp3/transcription Factor Staining Buffer Set (eBioscience) and further stained with anti-Foxp3-PE. Samples were subsequently analyzed by FACSLyric^TM^ (BD Biosciences).

### 4.5. CFSE Dilution Assay

Freshly isolated PBMCs or CD8-depleted PBMCs from HLA-A*02-positive healthy donors were labeled with 10 μM CFSE for 5 min and cultured for 14 days in the presence of Melan-A peptide as described above. On days 7, 9, 11, and 14, the cultures were stained with pMelan-PE and anti-CD8-APC to detect Melan-A-specific CD8^+^ T cells or intracellularly stained with anti-Foxp3-PE as described above following the surface staining with anti-CD4-PE-Cy5.

### 4.6. Rapid Expansion of Melan-A-Specific CD8^+^ T Cells and CD4^+^CD25^+^ Treg Cells

Melan-A-specific CD8^+^ T cells were expanded from PBMCs from HLA-A*02-positive healthy donors as described above. Ten days later, PBMCs were stained with pMelan-PE and anti-CD8-APC, and pMelan^+^CD8^+^ T cells were sorted using FACSAria (BD Biosciences). To prepare activated Treg cells, PBMCs were cultured for 14 days in the presence of Melan-A peptide as described above. On day 14, the cells were stained with anti-CD25-PE-Cy5 and anti-CD4-APC, and CD4^+^CD25^High^ cells were sorted using FACSAria (BD Biosciences). The sorted cells were expanded by the rapid expansion method [26]. In brief, a half million of the sorted and live cells were mixed with 3% auto plasma, ×50 irradiated allogeneic PBMC, 1000 IU/mL rhIL-2, and 30 ng/mL of anti-CD3 mAb and cultured for 14 days by routinely adding the medium supplemented with 3% auto plasma and 1000 IU/mL rhIL-2. Percentages of Treg cells in the expanded CD4^+^CD25^High^ cells were assessed by intracellular staining of Foxp3 before and after the rapid expansion.

### 4.7. Transcriptome Analysis of Melan-A-Specific CD8^+^ T Cells in the Presence or Absence of Activated Treg Cells

The expanded Melan-A-specific CD8^+^ T cells were rested by maintaining the cells in CM-supplemented 100 IU/mL rhIL-2 for 5 days. The cells were extensively washed with RPMI1640 medium to remove dead cells, labeled with 10 μM CFSE for 5 min, and mixed with 0, 25, and 50% of the expanded and activated Treg cells. For the Treg-mediated suppression assay, the mixture was cultured for 5 days in the presence of 0.5 μg/mL of anti-CD3 mAb and stained with anti-CD8-APC. Proliferation of Melan-A-specific CD8^+^ T cells and suppression capability of the expanded Treg cells were determined by assessing the CFSE dilution rate of Melan-A-specific CD8^+^ T cells. For transcriptome analysis of Melan-A-specific CD8^+^ T cells, CD8^+^ T cells were isolated from the mixture of Melan-A-specific CD8^+^ T cells and the expanded Treg cells 2 days after the culture. Total RNAs were extracted from the isolated CD8^+^ T cells and performed RNA sequencing (MACROGEN, Seoul, Republic of Korea).

### 4.8. Statistical Analysis

All data were analyzed with the statistical program Prism GraphPad 9.0 (San Diego, CA, USA). Student’s *t*-test was used to determine the statistical significance of differences.

## Data Availability

The RNA-seq datasets presented in this article are not readily available because the inclusion of sensitive genetic information from healthy volunteers and the written consent obtained from the participants does not fully cover the public release of these data. Requests to access the datasets should be directed to the corresponding author, B.K.C. (magetin@gmail.com).

## Supplementary Materials

The following supporting information can be downloaded at: https://www.mdpi.com/article/10.3390/ijms241813672/s1.
