# Supplementary material for: Cross-Activation of Regulatory T Cells by Self Antigens Limits Self-Reactive and Activated CD8+ T Cell Responses
Source: Int J Mol Sci. 2023 Sep 5;24(18):13672. doi: 10.3390/ijms241813672 (PMC10530955; doi:10.3390/ijms241813672)
Supplement: Supplementary file 1 [file ijms-24-13672-s001.zip › TalkFile_Supplementary Figure 1 and 2.pdf.pdf]

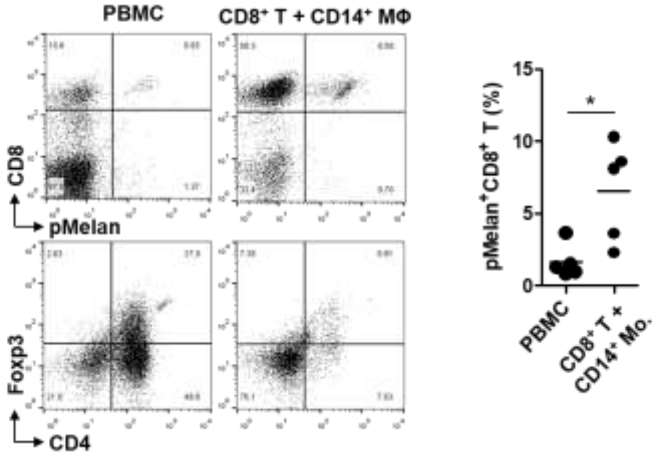

Supplemental Figure 1. Expansion of pMelan⁺CD8⁺ T cells in the absence of CD4⁺ T cells.

CD8⁺ T cells and CD14⁺ monocytes were isolated from PBMCs using CD8-microbeads or CD14-microbeads, respectively. The isolated CD8⁺ T cells were then mixed with the isolated monocytes at a ratio of 10:1. PBMCs and the isolated CD8⁺ T cells mixed with 10% monocytes were cultured with the Melan-A peptide for 14 days as described previously. On day 14, the cultured cells were stained with anti-CD8 antibody and A2/Melan-A_{25-36} multimer. To detect Treg cells, the cells were first stained with anti-CD4 antibody and subsequently intracellularly stained with anti-Foxp3 antibody. Statistical analysis was performed using Student's t-test, and the results are presented as means ± SDs (*$p < 0.05$).

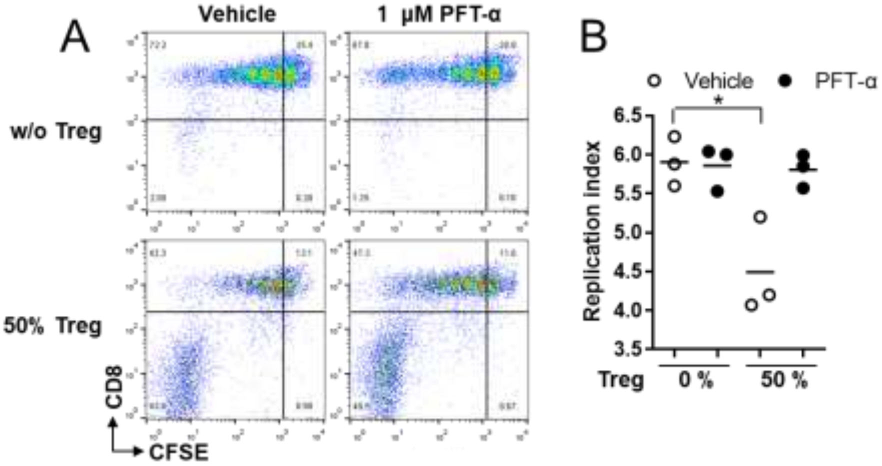

Supplemental Figure 2. Pifithrin-α hydrobromide restores Treg-mediated suppression of activated pMelan$^+$CD8$^+$ T cells.

(A) Treg cells and pMelan$^+$CD8$^+$ T cells were prepared from a single donor as described in Fig. 4. CFSE-labeled pMelan$^+$CD8$^+$ T cells were cultured in the presence of 0.5 μg/ml of anti-CD3 mAb with or without 50% of Treg cells for 5 days. The cultured cells were stained with anti-CD8-PE-Cy5 and subsequently analyzed by FACSCalbur (BD Biosciences). (B) Replication index of the dividing pMelan$^+$CD8$^+$ T cells was calculated using FlowJo software (Tree Star, Ashland, OR). Result is from a single donor with triplicated samples (n=3). Student's *t* test was performed in B (*$p < 0.05$).
