# Supplementary material for: Cross-Activation of Regulatory T Cells by Self Antigens Limits Self-Reactive and Activated CD8+ T Cell Responses
Source: Int J Mol Sci. 2023 Sep 5;24(18):13672. doi: 10.3390/ijms241813672 (PMC10530955; doi:10.3390/ijms241813672)
Supplement: Supplementary file 1 [file ijms-24-13672-s001.zip › TalkFile_Supplementary Table 1 and 2.pdf.pdf]

Supplementary Table 1. Amino acid sequence of MHC class I-restricted peptides from hTERT.

Name	Amino acid seq.	Name	Amino acid seq.
TERT #1	AAFRALVAQCL	TERT #20	YVPLLGSL
TERT #2	CLKELVARV	TERT #21	QTQLSRKLP
TERT #3	LAFGFALL	TERT #22	ALEAAANPAL
TERT #4	VGDDVLVH	TERT #23	ILAKFLHWL
TERT #5	FVLVAPSCA	TERT #24	RLVDDFLLV
TERT #6	GAATQARP	TERT #25	EARPALLTSRLRFIPK
TERT #7	SGTRHSH	TERT #26	RLFFYRKSV
TERT #8	KEQLRPSFLLSSLRPSL	TERT #27	YLFFYRKSV
TERT #9	PLFLELL	TERT #28	DLQVNSLQTV
TERT #10	AAVTPAA	TERT #29	YLQVNSLQTV
TERT #11	QSIGIRQ	TERT #30	GLLGASVLGL
TERT #12	IVNMDYV	TERT #31	ALLTSRLRFI
TERT #13	RPGLLGASV	TERT #32	RLTSRVKAL
TERT #14	TLTDLQP	TERT #33	TYVPLLGSL
TERT #15	LLCSLCYG	TERT #34	CYGDMENKL
TERT #16	LVRGVPEYGCVVNLR	TERT #35	AYQVCGPP
TERT #17	YSSYARTSIRASL	TERT #36	VYGFVRACL
TERT #18	IYKILLLQAY	TERT #37	VYAETKHFL
TERT #19	LGAKGAA	TERT #38	DYVVGARTF

Supplementary Table 2. Amino acid sequence of MHC class I-restricted peptides from WT-1.

Name	Amino acid seq.	Name	Amino acid seq.
WT-1 #1	ALLPAVPSL	WT-1 #11	AFTVHFSGQF
WT-1 #2	DLNALLPAV	WT-1 #12	RWPSCQKKF
WT-1 #3	SLGEQQYSV	WT-1 #13	RVPGVAPTL
WT-1 #4	RMFPNAPYL	WT-1 #14	DFKDCERRF
WT-1 #5	GVFRGIQDV	WT-1 #15	RTPYSSDNL
WT-1 #6	CMTWNQMNL	WT-1 #16	TSEKPFSCR
WT-1 #7	SGQFTGTAGA	WT-1 #17	FSRSDQLKR
WT-1 #8	VLDFAPPGA	WT-1 #18	LSHLQMHSR
WT-1 #9	AYPGCNKRYF	WT-1 #19	YMFPNAPYL
WT-1 #10	QYRIHTHGVF	WT-1 #20	CYTWNQMNL
